# Corticosterone primes the neuroinflammatory response to Gulf War Illness‐relevant organophosphates independently of acetylcholinesterase inhibition

**DOI:** 10.1111/jnc.14071

**Published:** 2017-06-14

**Authors:** Alicia R. Locker, Lindsay T. Michalovicz, Kimberly A. Kelly, Julie V. Miller, Diane B. Miller, James P. O'Callaghan

**Affiliations:** ^1^ Health Effects Laboratory Division Centers for Disease Control and Prevention National Institute for Occupational Safety and Health Morgantown West Virginia USA

**Keywords:** chlorpyrifos, diisopropyl fluorophosphate, neuroinflammation, physostigmine, pyridostigmine bromide, STAT3

## Abstract

Gulf War Illness (GWI) is a chronic multi‐symptom disorder affecting veterans of the 1991 Gulf War. Among the symptoms of GWI are those associated with sickness behavior, observations suggestive of underlying neuroinflammation. We have shown that exposure of mice to the stress hormone, corticosterone (CORT), and to diisopropyl fluorophosphate (DFP), as a nerve agent mimic, results in marked neuroinflammation, findings consistent with a stress/neuroimmune basis of GWI. Here, we examined the contribution of irreversible and reversible acetylcholinesterase (AChE) inhibitors to neuroinflammation in our mouse model of GWI. Male C57BL/6J mice received 4 days of CORT (400 mg/L) in the drinking water followed by a single dose of chlorpyrifos oxon (CPO; 8 mg/kg, i.p.), DFP (4 mg/kg, i.p.), pyridostigmine bromide (PB; 3 mg/kg, i.p.), or physostigmine (PHY; 0.5 mg/kg, i.p.). CPO and DFP alone caused cortical and hippocampal neuroinflammation assessed by qPCR of tumor necrosis factor‐alpha, IL‐6, C–C chemokine ligand 2, IL‐1β, leukemia inhibitory factor and oncostatin M; CORT pretreatment markedly augmented these effects. Additionally, CORT exposure prior to DFP or CPO enhanced activation of the neuroinflammation signal transducer, signal transducer and activator of transcription 3 (STAT3). In contrast, PHY or PB alone or with CORT pretreatment did not produce neuroinflammation or STAT3 activation. While all of the CNS‐acting AChE inhibitors (DFP, CPO, and PHY) decreased brain AChE activity, CORT pretreatment abrogated these effects for the irreversible inhibitors. Taken together, these findings suggest that irreversible AChE inhibitor‐induced neuroinflammation and particularly its exacerbation by CORT, result from non‐cholinergic effects of these compounds, pointing potentially to organophosphorylation of other neuroimmune targets.

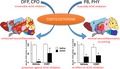

**Abbreviations used**
Ach: acetylcholine
AChE: acetylcholinesterase
BBB: blood‐brain barrier
CCL2: (C–C) chemokine ligand 2
CNS: central nervous system
CORT: corticosterone
CPF: chlorpyrifos
CPO: chlorpyrifos‐oxon
DFP: diisopropyl fluorophosphate
GFAP: glial fibrillary acidic protein
GWI: Gulf War Illness
IL‐1β: interleukin 1 beta
IL‐6: interleukin 6
JAK: Janus kinase
LIF: leukemia inhibitory factor
OP: organophosphate
OSM: oncostatin M
PB: pyridostigmine bromide
PHY: physostigmine
pSTAT3^Tyr705^: phosphorylated STAT3 tyrosine 705
SDS: sodium dodecyl sulfate
STAT3: signal transducer and activator of transcription 3
TNFα: tumor necrosis factor‐alpha

Approximately 200 000 soldiers that served in the 1991 Persian Gulf War returned with clinical symptoms that include chronic fatigue, headache, cognitive impairment, depression, muscle and joint pain, and gastrointestinal issues, among others. This multi‐symptom illness has been termed Gulf War Illness (GWI) (Steele 2000; Golomb 2008; Research Advisory Committee (RAC) on Gulf War Veterans’ Illnesses 2013; Heng 2016; White *et al*. 2016). The set of symptoms that characterizes GWI closely resembles the symptoms of protracted ‘sickness behavior’ (e.g., fatigue, nausea, sleep disturbances, cognitive impairments), a condition that is accompanied by a robust neuroinflammatory response seen in both humans and animal models (Dantzer *et al*. 2008; O'Callaghan *et al*. 2015). While a chronic or heightened neuroinflammatory response is linked to the symptoms of GWI in veterans, the underlying causes of this response have not been fully elucidated.

Soldiers that served in the Gulf War were exposed to a number of acetylcholinesterase (AChE) inhibitors. The irreversible AChE inhibitor, chlorpyrifos (CPF), was sprayed on uniforms and used in living quarters as an insecticide (Research Advisory Committee (RAC) on Gulf War Veterans’ Illnesses 2008); another irreversible AChE inhibitor, dichlorvos, was used in ‘pest strips’ placed in encampments (Research Advisory Committee (RAC) on Gulf War Veterans’ Illnesses 2008). Soldiers also were potentially exposed to another irreversible AChE inhibitor, the nerve agent, sarin, likely from a downwind plume following the demolition of munitions at several sites, notably, the Khamisiyah Ammunition Storage Facility (Research Advisory Committee (RAC) on Gulf War Veterans’ Illnesses 2008; White *et al*. 2016). Finally, soldiers self‐administered the reversible AChE inhibitor, pyridostigmine bromide (PB), which was consumed as a prophylactic treatment against potential nerve agent exposure (Tuovinen *et al*. 1999; Research Advisory Committee (RAC) on Gulf War Veterans’ Illnesses 2008; White *et al*. 2016). The shared actions of these irreversible and reversible AChE inhibitors suggests that exposure to these compounds in theater may have contributed to the symptoms of Gulf War Illness (Fukuda *et al*. 1998; Cao *et al*. 2011; Patocka *et al*. 2015), specifically by increasing acetylcholine (ACh) as a result of the inhibition of AChE (Friedman *et al*. 1996; Golomb 2008).

While inhibition of AChE by GWI‐relevant compounds serves as an attractive hypothesis for a contributory role of ACh in the development of GWI, other observations would argue against such a role. For example, AChE inhibition and the resulting increase in ACh levels should make these compounds anti‐inflammatory agents caused by activation of the ‘cholinergic anti‐inflammatory pathway’ (Pavlov *et al*. 2003; Pavlov and Tracey 2005), that is, effects that contrast with the known proinflammatory actions observed for organophosphates (OPs) in mouse and rat models (Spradling *et al*. 2011; O'Callaghan *et al*. 2015). The disparate potential roles for ACh in neuroinflammation make it seem possible that cholinergic mechanisms, and inhibition of AChE in particular, may not be responsible for symptoms associated with GWI. One way to address the role of AChE inhibition in GWI would be to assay the activity of AChE and the expression of proinflammatory mediators in samples obtained from animals exposed to GWI‐relevant compounds and conditions. This approach would allow for a comparison among irreversible and reversible AChE inhibitors implicated in GWI with respect to their ability to cause neuroinflammation and inhibition of AChE.

Previously, we developed a mouse model of GWI that utilizes diisopropyl fluorophosphate (DFP) as a sarin surrogate and exogenous corticosterone (CORT) at levels associated with high physiological stress (Sapolsky *et al*. 1985) to replicate GW theater conditions (O'Callaghan *et al*. 2015). We found that exposure to DFP, an irreversible inhibitor of AChE, results in a brain‐wide neuroinflammation that, paradoxically, is markedly augmented by prior exposure to the anti‐inflammatory glucocorticoid, CORT. Here, we tested the hypothesis that AChE inhibition was not required for expression of neuroinflammatory mediators using our previously developed mouse model of GWI (O'Callaghan *et al*. 2015). We found that irreversible, but not reversible inhibition of AChE, was associated with neuroinflammation, effects enhanced by prior exposure to high physiological levels of CORT. Consistent with these findings, the downstream signaling effector of neuroinflammation, phosphorylated signal transducer and activator of transcription 3 tyrosine 705 (pSTAT3^Tyr705^) (O'Callaghan *et al*. 2014) was activated by irreversible, but not reversible, inhibitors of AChE and was also enhanced by prior exposure to CORT. These findings indicate that the CORT‐primed neuroinflammation associated with GWI‐related AChE inhibitors is unlikely to be directly induced by AChE inhibition.

## Methods

### Materials

Drugs and chemicals were obtained from the following sources: chlorpyrifos oxon (CPO; Chem Service, Inc., West Chester, PA, USA), DFP (Sigma‐Aldrich Co., St. Louis, MO, USA), PB (Sigma‐Aldrich Co.), physostigmine (PHY; Sigma‐Aldrich Co.), ethanol (Sigma‐Aldrich Co.), CORT (Steraloids Inc., Newport, RI, USA), 5,5‐dithio‐bis‐(2‐nitrobenzoic acid) (Sigma‐Aldrich Co.), tetraisopropyl pyrophosphoramide (Sigma‐Aldrich Co.), and acetylthiocholine iodide (Sigma‐Aldrich Co.). Rabbit Anti‐phospho STAT3^tyr705^ antibodies were obtained from Cell Signaling, Inc. (RRID: AB_621843; Beverly, MA, USA). The materials used in glial fibrillary acidic protein (GFAP) ELISA previously have been described in detail (O'Callaghan *et al*. 1991; O'Callaghan 2002). Material used for additional tissue analyses were of at least analytical grade and purchased from various commercial sources.

### Animals

Adult male (8–12 weeks of age; weighing approximately 22 g) C57BL/6J mice were purchased from Jackson Labs (RRID: IMSR_JAX:000664; Bar Harbor, ME, USA). Upon arrival, mice were individually housed in a temperature‐ (21 ± 1°C) and humidity‐controlled (50 ± 10%) colony room that was maintained under filtered positive‐pressure ventilation on a 12 h light (0600 EDT)/12 h dark cycle (1800 EDT). The plastic cages were 46 cm in length by 25 cm in width by 15 cm in height; cage bedding consisted of heat‐treated pine shavings spread at a depth of 4 cm. Mice were single housed and given *ad libitum* access to food (Harlan 7913 irradiated NIH‐31 modified 6% rodent chow) and water. All mouse procedures were performed according to protocols approved by the Institutional Animal Care and Use Committee of the Centers for Disease Control and Prevention, National Institute for Occupational Safety and Health, and the animal colony was certified by the Association for Assessment and Accreditation of Laboratory Animal Care International (AAALAC).

### Dosing

Mice (*n* = at least four/group, animals were arbitrarily assigned to groups by the experimenter) were given intraperitoneal (i.p.) injections of saline (0.9%), peanut oil (CPO vehicle), CPO (8 mg/kg,) DFP (4 mg/kg), PB (3 mg/kg) or PHY (0.5 mg/kg) in the morning and returned to their home cage (experimenter was not blinded). The doses of AChE inhibitors were selected based on their ability to produce the symptoms of cholinergic crisis [e.g., SLUD (salivation, lacrimation, urination, and defecation) and seizures] while displaying mortality below LD25. CORT was given in the drinking water (400 mg/L in 1.2% EtOH) for 4 days prior to AChE inhibitor or vehicle exposure. This regimen of CORT was chosen because of its ability to achieve high circulating levels of this hormone, similar to those achieved with repeated stress (Ganon and McEwen 1990), and because it was capable of producing significant immunosuppression as evidenced by involution of the thymus (O'Callaghan *et al*. 1991). Here, average thymus weights were significantly reduced from 45.4 ± 2.7 mg in vehicle‐treated to 12.8 ± 1.8 mg in CORT‐treated mice with average body weight of 21.5 ± 0.4 g. This study used twice the CORT concentration in the drinking water, but employed a 4‐day instead of a 7‐day CORT exposure regimen in comparison to the CORT regimen used in our original mouse model of GWI (O'Callaghan *et al*. 2015). Mice were killed by decapitation at 30 min (for AChE activity assay) or 6 h (for all other analyses) post‐AChE or vehicle injection. No differences were seen for the endpoints evaluated between mice exposed to peanut oil and saline (data not shown); therefore, peanut oil alone groups were excluded from analyses.

### Brain dissection and preparation

Immediately after decapitation, whole brains were removed from the skull and the hippocampus and cortex were dissected free‐hand on a thermoelectric cold plate (Model TCP‐2; Aldrich Chemical Co., Milwaukee, WI, USA) using fine curved forceps (Roboz, Washington, DC, USA). Brain regions from one side of the brain were frozen and stored at −80°C until subsequent isolation of total RNA. Brain regions from the other side of the brain were weighed and then homogenized with the aid of a sonic probe (model XL‐2005; Heat Systems, Farmingdale, NY, USA) in 10 volumes of hot (90–95°C) 1% sodium dodecyl sulfate (SDS). This tissue was then stored at −80°C until total protein assay and immunoassays of GFAP and immunoblots of pSTAT3^tyr705^ were conducted. For the AChE activity assay, brains were bifurcated and one half of the brain was frozen and stored at −80°C until analysis.

### RNA isolation, cDNA synthesis, and qPCR

The total RNA from the hippocampus and cortex were isolated using Trizol^®^ reagent (Thermo Fisher Scientific, Waltham, MA, USA) and Phase‐lock heavy gel (Eppendorf, AG Hamburg, Germany), and purified using RNeasy mini‐spin columns (Qiagen, Valencia, CA, USA). Total RNA (1 μg) was reverse transcribed to cDNA using Superscript III and oligo (dT)_12–18_ primers (Thermo Fisher Scientific) in a 20 μL reaction. Real‐time PCR analysis of glyceraldehyde‐3‐phosphate dehydrogenase (endogenous control), tumor necrosis factor‐alpha (TNFα), C–C chemokine ligand 2, leukemia inhibitor factor, interleukin 6 (IL‐6), interleukin 1beta (IL‐1β), oncostatin M and GFAP was performed using an Applied Biosystems 7500 real‐time PCR system (Thermo Fisher Scientific) in combination with TaqMan^®^ chemistry. All PCR amplifications (40 cycles) were performed in a total volume of 50 μL, containing 1 μL cDNA, 2.5 μL of the specific Assay of Demand primer/probe mix (Thermo Fisher Scientific), and 25 μL of Taqman^®^ Universal master mix (Thermo Fisher Scientific). Sequence detection software (version 1.7; Applied Biosystems/Thermo Fisher Scientific) results were exported into Excel for further analysis. Relative quantification of gene expression was performed using the comparative threshold (ΔΔC_T_) method. Changes in mRNA expression levels were calculated after normalization to glyceraldehyde‐3‐phosphate dehydrogenase. The ratios obtained after normalization are expressed as fold changes over corresponding controls.

### pSTAT3 immunoblot analysis

Activation of the Janus kinase (JAK)‐STAT3 neuroinflammation effector pathway (O'Callaghan *et al*. 2014) was assessed by quantifying pSTAT3^tyr705^ from immunoblots of tissue homogenates, with detection of fluorescent signals using an infrared fluorescence scanner (Licor Biosciences; Lincoln, NE, USA) as previously described (Sriram *et al*. 2004; Dinapoli *et al*. 2010; O'Callaghan *et al*. 2014). Briefly, following incubation with primary antibodies (rabbit anti‐phospho‐STAT3^tyr705^[1 : 500]; RRID: AB_331586; Cell Signaling, Danvers, MA, USA), blots were washed with phosphate buffered saline with 0.1% Tween‐20 and incubated with anti‐rabbit fluorescent‐labeled secondary antibody (1 : 2500; RRID: AB_621843) for 1 h prior to scanning by Licor. We note the general requirement for using focused microwave irradiation sacrifice to preserve steady‐state *in vivo* phosphorylation does not apply in the case of pSTAT3^tyr705^ (O'Callaghan and Sriram 2004).

### Immunoassay of GFAP

GFAP was assayed in accordance with a previously described procedure (O'Callaghan 1991, 2002). In brief, a rabbit polyclonal antibody to GFAP (1 : 400; RRID: AB_10013382; DAKO, Carpenteria, CA, USA) was coated on the wells of Immulon‐2 microtiter plates (Thermo Labsystems, Franklin, MA, USA). The SDS homogenates and standards were diluted in phosphate‐buffered saline (pH 7.4) containing 0.5% Triton X‐100. Standards consisted of SDS homogenates of hippocampus with known concentration of GFAP and were prepared the same way as the samples. After blocking non‐specific binding with 5% non‐fat dairy milk, aliquots of the homogenate and standards were added to the wells and incubated. Following washes, a mouse monoclonal antibody to GFAP (1 : 250; RRID: AB_477010; Sigma‐Aldrich Co.) was added to ‘sandwich’ the GFAP between the two antibodies. An alkaline phosphatase‐conjugated antibody directed against mouse IgG (1 : 2000; RRID: AB_2340075; Jackson ImmunoResearch Labs, West Grove, PA, USA) was then added and a colored reaction product was obtained by subsequent addition of the enzyme substrate, p‐nitrophenol. Quantification was achieved by spectrophotometry of the colored reaction product at 405 nm in a microplate reader, Spectra Max Plus, and analyzed using Soft Max Pro Plus software (Molecular Devices, Sunnyvale, CA, USA). The amount of GFAP in the samples was calculated as micrograms of GFAP per milligram total protein.

### Acetylcholinesterase activity

Acetylcholinesterase activity was assessed via a protocol adapted from the Ellman method (Ellman *et al*. 1961; Lein and Fryer 2005). Briefly, one frozen cerebral hemisphere was homogenized with a sonic probe (mode XL‐2005; Heat Systems) in 10 volumes of sodium phosphate buffer (0.1 M, pH 8.0) with 1% Triton X‐100. Immediately following homogenization, the brains were centrifuged at 13 400 × g and the supernatant was removed and diluted 1 : 10 with sodium phosphate buffer prior to analysis. Following addition of a 5,5‐dithio‐bis‐(2‐nitrobenzoic acid)/tetraisopropyl pyrophosphoramide solution, samples were incubated for 5 min and reaction began when acetylthiocholine iodide was added. Quantification was achieved by spectrophotometry of the colored reaction product at 405 nm over a 10 min kinetic assay (16 cycles) in a Spectra Max Plus microplate reader and analyzed using Soft Max Pro Plus software (Molecular Devices). Acetylcholinesterase activity (μM substrate formed/min/mg total protein) was calculated based on the amount of total protein determined using the Pierce™ BCA Protein Assay kit (Thermo Fisher Scientific), per manufacturer's instructions. Acetylcholinesterase activity was normalized to saline control and is reported as a percentage.

### Statistics

For calculation of sample size, anova power analysis was performed using SigmaPlot (Systat Software, Inc., San Jose, CA, USA; RRID:SCR_003210; v12.5) using previously obtained mean differences and standard deviations between treated and control tissue with a power of 0.8 and α = 0.05; the sample size was estimated at four mice per group. Larger sample sizes of 5–7 were utilized to control for AChE inhibition induced mortality and endpoint variability [removal of outliers via Grubbs’ test (α = 0.05)] to achieve final sample sizes of at least *n* = 4 per group. All statistical analyses were performed using SigmaPlot. Two‐way anovas were conducted on log transformed values with Fisher least significant difference *post‐hoc* tests. Statistical significance was set at α = 0.05 (*p* < 0.05), and all graphs show mean ± SEM of raw values, unless otherwise stated.

## Results

### Irreversible AChE inhibitors produce neuroinflammation that is markedly enhanced by CORT pretreatment

**Figure 1:**
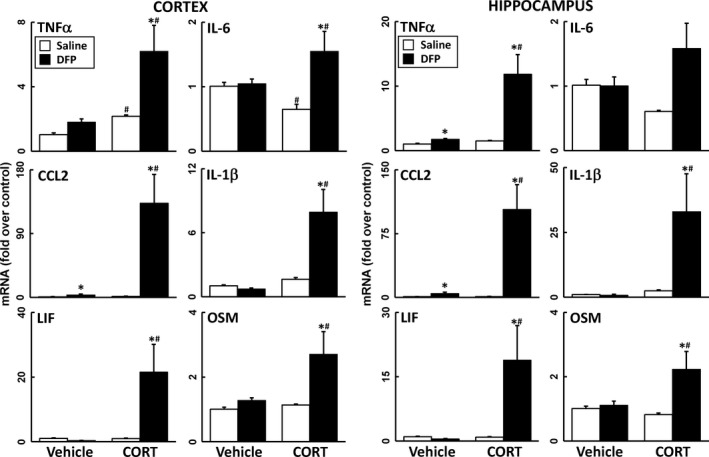
Corticosterone (CORT) pretreatment exacerbates diisopropyl fluorophosphate (DFP)‐induced neuroinflammation. Effects of DFP exposure (4 mg/kg, i.p.) with and without prior CORT treatment (400 mg/L, 1.2% EtOH) on neuroinflammation as measured by qPCR of inflammatory cytokines and chemokines at 6 h post‐DFP. Tumor necrosis factor‐alpha (TNFα), IL‐6, (C–C) chemokine ligand 2 (CCL2), IL‐1b, leukemia inhibitory factor (LIF), and oncostatin M (OSM) were measured in cortex (left panels) and hippocampus (right panels). Data represents mean ± SEM (*N* = 4–6 mice/group). Statistical significance of at least *p* ≤ 0.05 is denoted by * compared to relevant control (vehicle or CORT) and # compared within treatment (saline or DFP).

**Figure 2:**
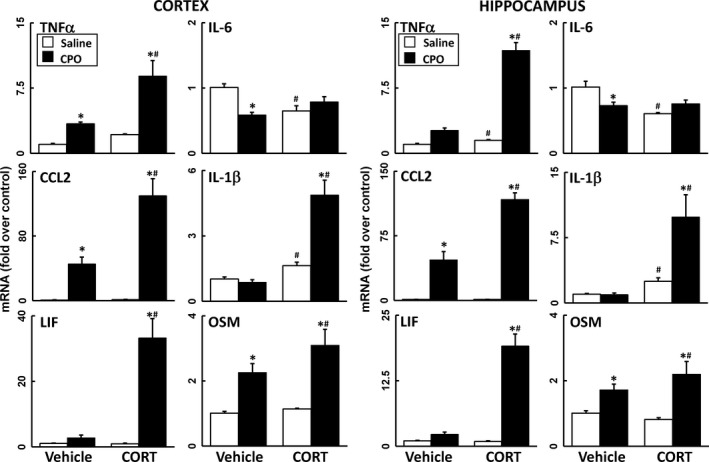
Corticosterone (CORT) pretreatment exacerbates chlorpyrifos oxon (CPO)‐induced neuroinflammation. Effects of CPO exposure (8 mg/kg, i.p.) with and without prior CORT treatment (400 mg/L, 1.2% EtOH) on neuroinflammation as measured by qPCR of inflammatory cytokines and chemokines at 6 h post‐CPO. Tumor necrosis factor‐alpha (TNFα), IL‐6, (C–C) chemokine ligand 2 (CCL2), IL‐1b, leukemia inhibitory factor (LIF), and oncostatin M (OSM) were measured in cortex (left panels) and hippocampus (right panels). Data represents mean ± SEM (*N* = 4–6 mice/group). Statistical significance of at least *p* ≤ 0.05 is denoted by * compared with relevant control (vehicle or CORT) and # compared with treatment (saline or CPO).

Administration of the irreversible AChE inhibitor, DFP (used as a sarin surrogate), resulted in an increased expression of CCL2 and TNFα in cortex and or hippocampus (Fig. 1). Prior treatment with CORT in the drinking water for 4 days resulted in significant increases in all six cytokines/chemokines in both brain regions (Fig. 1) (with the exception of IL‐6 in hippocampus). Small increases (TNFα) or decreases (IL‐6) also were observed for the CORT alone condition in cortex. The findings for DFP were extended to another irreversible inhibitor of AChE, CPO (the oxon metabolite of the insecticide CPF). Like DFP, administration of CPO alone produced a significant increase in the expression of some proinflammatory mediators in cortex and hippocampus (Fig. 2) (and a small decrease in expression of IL‐6 in hippocampus). Also consistent with the data for DFP, CPO‐induced neuroinflammation was markedly increased by prior treatment with CORT, with the exception of mRNA for IL‐6 (Fig. 2). These results act to expand our DFP‐based GWI model (O'Callaghan *et al*. 2015) to include another GW‐relevant exposure, chlorpyrifos.

### Reversible AChE inhibition does not produce neuroinflammation

**Figure 3:**
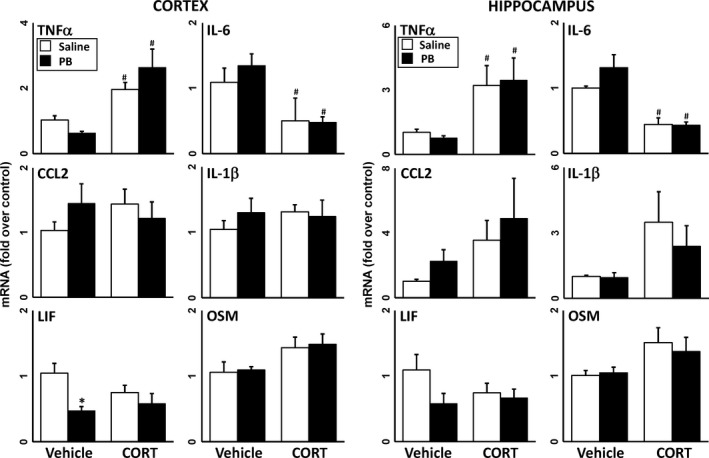
Pyridostigmine bromide (PB) does not cause significant neuroinflammation with or without prior corticosterone (CORT) exposure. Effects of PB exposure (3 mg/kg, i.p.) with and without prior CORT treatment (400 mg/L, 1.2% EtOH) on neuroinflammation as measured by qPCR of inflammatory cytokines and chemokines at 6 h post‐PB. Tumor necrosis factor‐alpha (TNFα), IL‐6, (C–C) chemokine ligand 2 (CCL2), IL‐1b, leukemia inhibitory factor (LIF), and oncostatin M (OSM) were measured in cortex (left panels) and hippocampus (right panels). Data represents mean ± SEM (*N* = 4–6 mice/group). Statistical significance of at least *p* ≤ 0.05 is denoted by * compared with relevant control (vehicle or CORT) and # compared with treatment (saline or PB).

**Figure 4:**
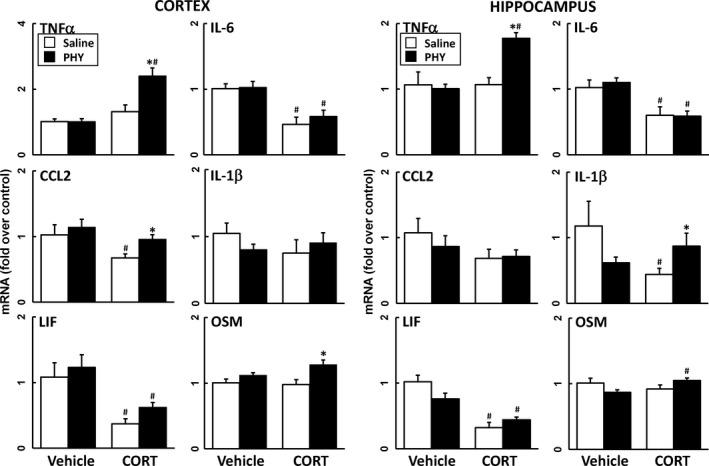
The brain penetrant AChE inhibitior, physostigmine (PHY), has little effect on neuroinflammation in the presence of corticosterone (CORT) pretreatment. Effects of PHY exposure (0.5 mg/kg, i.p.) with and without prior CORT treatment (400 mg/L, 1.2% EtOH) on neuroinflammation as measured by qPCR of inflammatory cytokines and chemokines at 6 h post‐PHY. Tumor necrosis factor‐alpha (TNFα), IL‐6, (C–C) chemokine ligand 2 (CCL2), IL‐1b, leukemia inhibitory factor (LIF), and oncostatin M (OSM) were measured in cortex (left panels) and hippocampus (right panels). Data represents mean ± SEM (*N* = 4–6 mice/group). Statistical significance of at least *p* ≤ 0.05 is denoted by * compared with relevant control (vehicle or CORT) and # compared with treatment (saline or PHY).

Epidemiologic studies have associated the reversible AChE inhibitor, PB, a drug given as a prophylactic measure against nerve agent exposure during the Gulf War, with the symptomology of GWI (Steele *et al*. 2012; White *et al*. 2016). PB does not readily cross the blood‐brain barrier (BBB) (Rice *et al*. 1997; Tuovinen *et al*. 1999; Song *et al*. 2002; Amourette *et al*. 2009); therefore, to assess the potential for reversible AChE inhibition in the central nervous system (CNS) to produce neuroinflammation, mice were exposed to PB or the CNS‐penetrant reversible AChE inhibitor, PHY, with or without CORT pretreatment. In general, neither agent produced significant neuroinflammation alone or with prior CORT treatment, except for a minor effect on TNFα (Figs 3 and 4). In addition, reversible AChE inhibitors tend to be anti‐inflammatory, reducing neuroinflammation significantly below control levels in some cases (Figs 3 and 4).

### CORT pretreatment of irreversible (CPO and DFP) but not reversible (PHY and PB) AChE inhibitors produces STAT3 Activation

**Figure 5:**
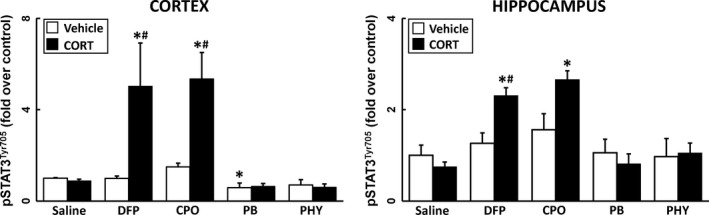
Prior corticosterone (CORT) treatment significantly increases phosphorylated signal transducer and activator of transcription 3 tyrosine 705 (pSTAT3^tyr705^) levels in diisopropyl fluorophosphate (DFP) and chlorpyrifos oxon (CPO) treated mice. Effects of CORT pretreatment (400 mg/L, 1.2% EtOH) on the phosphorylation of STAT3 at 6 h following AChE inhibitor exposure. pSTAT3^tyr705^ protein was measured in the cortex and hippocampus of saline, DFP, CPO, Pyridostigmine bromide (PB), and physostigmine (PHY) treated mice. Data represents mean ± SEM (*N* = 4–6 mice/group). Statistical significance of at least *p* ≤ 0.05 is denoted by * compared with relevant control (vehicle or CORT) and # compared within treatment (saline or AChE inhibitor).

Proinflammatory cytokine signaling can activate the JAK/STAT3 pathway, as evidenced by the pSTAT3^tyr705^ (O'Callaghan *et al*. 2014). Here, we observed an increase in pSTAT3^Tyr705^ in the cortex and hippocampus of CORT+DFP and CORT+CPO exposed mice, an effect not seen following CORT+PB or CORT+PHY exposure (Fig. 5). This observation is consistent with the enhanced neuroinflammation induced by DFP and CPO following CORT pretreatment (Figs 1 and 2). Activation of the STAT3 pathway in the absence of astrogliosis, as is the case for exposure to DFP and CPO in our model (see below and O'Callaghan *et al*. 2015), is suggestive of the activation of microglia (O'Callaghan *et al*. 2014).

### DFP, CPO and PHY, but not PB, inhibit brain acetylcholinesterase activity, but prior CORT pretreatment reduces the inhibition caused by DFP and CPO

**Figure 6:**
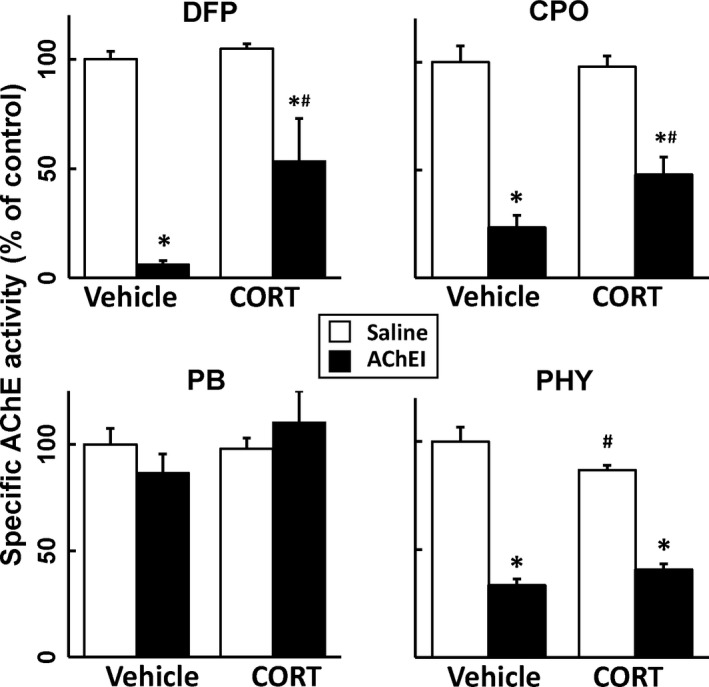
Neuroactive AChE inhibitors significantly reduce AChE activity in the brain, an effect that is reversed by corticosterone (CORT) diisopropyl fluorophosphate (DFP) and CORT chlorpyrifos oxon (CPO). AChE activity was measured in one brain hemisphere 30 min following AChE inhibitor exposure [DFP, CPO, pyridostigmine bromide (PB), and physostigmine (PHY)] with or without CORT pretreatment (400 mg/L, 1.2% EtOH). Data represents mean % ± SEM (*N* = 4–6 mice/group). Statistical significance of at least *p* ≤ 0.05 is denoted by * compared with relevant control (vehicle or CORT) and # compared with treatment (saline or AChE inhibitor).

One hypothesis regarding AChE inhibitors and the development of GWI is that cholinergic effects of these compounds have made lasting physiological impacts that may contribute to the underlying cause of GWI (Golomb 2008). To evaluate this theory, the AChE enzyme activity was measured in mice treated with both irreversible and reversible AChE inhibitors with or without prior CORT treatment. As expected, DFP, CPO, and PHY exposure resulted in significant inhibition of enzyme activity 30 min after treatment and PB had no effect on AChE activity in the brain (Fig. 6). Interestingly, pretreatment with CORT significantly ‘recovered’ some of the AChE activity inhibited by DFP and CPO (Fig. 6). However, CORT pretreatment did not ameliorate PHY‐induced AChE inhibition (Fig. 6). Furthermore, despite claims that stressors or stress hormone may increase BBB permeability to PB (Friedman *et al*. 1996; Hanin 1996; Shen 1998), there was no effect of PB on AChE activity in the brain with or without prior CORT exposure (Fig. 6). The recovery of AChE activity with DFP and CPO following CORT pretreatment suggests that the enhanced neuroinflammation seen with these conditions is not dependent on a particular degree of AChE inhibition. Moreover, CORT treatment prior to DFP or CPO exposure brings AChE activity back to a level comparable with PHY exposure, which we have shown does not instigate neuroinflammation (Fig. 4).

### GFAP mRNA and protein levels were unchanged as a result of any AChE inhibitor exposure with or without CORT pretreatment

Damage to the CNS by any type of insult, including neurotoxicants, results in hypertrophy of astrocytes at sites of injury (O'Callaghan and Sriram 2005; O'Callaghan *et al*. 2008). Injury‐induced activation of astrocytes is associated with an accumulation of the astrocyte intermediate filament protein, GFAP (O'Callaghan and Sriram 2005; O'Callaghan *et al*. 2014). Thus, GFAP expression and levels can be used as a biomarker of underlying CNS damage. With minor exceptions, no changes in GFAP or GFAP mRNA were seen with any exposure condition (data not shown), results consistent with our prior findings for DFP with and without CORT (O'Callaghan *et al*. 2015), and findings suggestive of a lack of underlying damage as a result of any treatment.

## Discussion

GWI is a multisymptom disorder with characteristics of sickness behavior, a symptom profile known to be driven by underlying neuroinflammation (Steele 2000; Konsman *et al*. 2002; Dantzer *et al*. 2008; Bay‐Richter *et al*. 2011). Among the exposures implicated in the etiology of GWI are AChE inhibitors: the nerve agent, sarin, the insecticide, CPF, and the nerve agent prophylactic, PB. Here, we have shown that exposure to the irreversible AChE inhibitors, DFP (a sarin surrogate) and CPO (the oxon derivative of CPF), results in neuroinflammatory responses in both the cortex and hippocampus that are augmented by 4 days of prior exposure to CORT in the drinking water. The enhanced neuroinflammation prompted by prior CORT exposure was associated with a subsequent increase in activation of STAT3, a key down‐stream effector of neuroinflammation (O'Callaghan *et al*. 2014). The results obtained with DFP and CPO could not be extended to the reversible AChE inhibitors, PB and PHY. Moreover, the neuroinflammatory responses associated with exposure to DFP and CPO did not correlate with AChE inhibition, as the conditions that resulted in robust neuroinflammation (CORT+DFP and CORT+CPO) also blunted overall AChE inhibition. These observations suggest that the neuroinflammatory responses that may underlie the symptoms of GWI are not related to the cholinergic effects of these compounds in the CNS, as they are not generalizable across both irreversible and reversible inhibitors. However, neuroinflammation was instigated by both irreversible AChE inhibitors (DFP and CPO) that are also classified as OP compounds, suggesting that it may be the phosphorylating action of these chemicals that is the effector of GWI.

Our present findings extend our previously established GWI model (using DFP as a sarin surrogate) (O'Callaghan *et al*. 2015) to the GWI‐relevant pesticide, CPF. The augmented neuroinflammatory response seen with CORT administration prior to both DFP and CPO is paradoxical, because glucocorticoids, such as CORT, traditionally are considered to be immunosuppressants (Barnes 2006; Coutinho and Chapman 2011). The mechanism through which this CORT‐‘primed’ neuroinflammatory response is achieved is currently unknown, but our findings are consistent with other studies that have found similar pro‐inflammatory effects with stressor or stress hormones alone or in response to neuroinflammatory exposures (Johnson *et al*. 2003; O'Connor *et al*. 2003; Loram *et al*. 2011). CORT priming, however, does not occur with all neuroinflammatory exposures. For example, prior CORT administration in the drinking water does not enhance the inflammatory response observed after dopaminergic neurotoxicity caused by MPTP, despite enhancing the neuroinflammatory response to the dopaminergic neurotoxicant, METH (Kelly *et al*. 2012).

AChE inhibition does not appear to drive neuroinflammation observed in our GWI model. The irreversible inhibitors of AChE, DFP and CPO, and the brain penetrant reversible inhibitor of AChE, PHY, inhibited brain AChE activity as expected. Such effects likely do not underlie neuroinflammation, because inhibition of AChE by the reversible AChE inhibitor, PHY, did not induce neuroinflammation with or without prior CORT. Moreover, CORT‐enhanced neuroinflammation associated with exposure to DFP and CPO occurred despite a reduction in AChE inhibition by these compounds when given with CORT pretreatment. One of the theories regarding the initiation of GWI is that stressors precipitated adverse effects of PB, administered as a nerve agent prophylactic (Friedman *et al*. 1996; Research Advisory Committee (RAC) on Gulf War Veterans’ Illnesses 2008), potentially by allowing this compound to gain entry to the CNS. PB has a quaternary amine structure that should prevent BBB penetration and limit inhibition of AChE activity to the periphery (Rice *et al*. 1997; Tuovinen *et al*. 1999; Song *et al*. 2002; Amourette *et al*. 2009). Exposure to acute stressors/stress hormones (e.g., cortisol), however, can increase permeability of the BBB (Friedman *et al*. 1996; Esposito *et al*. 2002). Thus, several studies have suggested that symptoms of GWI result from the actions of a peripherally‐acting AChE inhibitor (i.e., PB) entering into the CNS, at least in part due to physiological stress‐induced permeability of the BBB (Friedman *et al*. 1996; Hanin 1996; Shen 1998). Our findings suggest that any effects of PB related to GWI, in our model, are not because of brain entry of this compound resulting from pretreatment with CORT, because brain AChE activity was not reduced following exposure to these two treatments. These findings are consistent with those from other studies where PB permeability changes were not found following exposure to high physiological levels of CORT (Park *et al*. 2008; Amourette *et al*. 2009). Furthermore, recent evidence has indicated that exogenous glucocorticoid exposure actually may serve to strengthen the BBB, restricting, rather than facilitating the movement of peripherally administered chemicals and biologics into the brain (Calabria *et al*. 2006; Chen *et al*. 2013; Furihata *et al*. 2015).

Our animal model‐based findings do more to rule out a role of AChE in GWI than they do to point to a specific non‐AChE mechanism. Nevertheless, our findings provide indirect support to numerous prior investigations that point to: disrupted axonal transport via covalent binding of OPs to tubulin (Prendergast *et al*. 2007), disruption of neurofilament protein function by aberrant phosphorylation (Abou‐Donia *et al*. 1988), reduced hippocampal neurogenesis (Parihar *et al*. 2013) and increases in amyloid precursor protein and Aβ levels (Sanchez‐Santed *et al*. 2016), all as potential (non‐AChE related) effects underlying symptoms exhibited by ill GW veterans. Beyond some of these ‘alternative’ targets of irreversible AChE inhibitors that have been implicated in GWI, many key pathways may contain additional vulnerable phospho‐substrates that, upon phosphorylation by OPs, may alter signal transduction in a manner resulting in the symptoms of GWI. Screening tools for phosphoprofiling assessments (Zhu *et al*. 2010; Singec *et al*. 2016; Tinti *et al*. 2017) can be implemented to discover and characterize novel targets of organophosphorylation by OPs in animal models with the overall goal of identifying drug‐able targets to treat GWI. While our present findings draw focus away from the AChE inhibiting activity of these compounds as a basis for the neuroinflammatory effects associated with GWI, our observations do not detract from a role of these AChE inhibiting exposures in GWI. Multiple epidemiological studies have implicated AChE inhibitors (pesticides, PB, nerve agent) in the etiology of symptoms associated with GWI (Research Advisory Committee (RAC) on Gulf War Veterans’ Illnesses 2013). For example, a recent study (Steele *et al*. 2015) showed that butyrylcholinesterase genotypes possessed by the most ill GW veterans may have resulted in lower metabolism and higher exposure levels of self‐administered PB. Such findings points to a role of genetic susceptibility in GWI and suggest the need to more broadly assess the potential diversity of targets underlying the disorder.

While neuroinflammatory responses often accompany neurotoxicant‐induced neuronal damage (Whittington *et al*. 1989; O'Callaghan and Jensen 1992; Kelly *et al*. 2012; O'Callaghan *et al*. 2014), including exposure to irreversible AChE inhibitors (Chapman *et al*. 2006; Stapleton and Chan 2009; Li *et al*. 2011; Lim *et al*. 2011; Banks and Lein 2012; Ferchmin *et al*. 2014), neuroinflammation can occur in the absence of neuronal damage (O'Callaghan *et al*. 2014). Our present findings are consistent with the induction of neuroinflammation in the absence of damage, because GFAP mRNA and GFAP were not increased, as would have been expected if underlying neural damage had elicited astrogliosis, a dominant cellular reaction following exposure to broad classes of known neurotoxic conditions (O'Callaghan and Sriram 2005).

The observed activation of STAT3, a transcription factor associated with both damage‐induced astrogliosis and neuroinflammation in the absence of neural damage and astrogliosis (O'Callaghan *et al*. 2014), was expected because CORT‐primed DFP‐ and CPO‐induced neuroinflammation, even in the absence of neural damage, would signal through activation of STAT3. Because STAT3 was activated, it is unlikely that enhanced expression of neuroinflammatory mediators occurred in the absence of an increase in the mediators themselves, as opposed to their mRNA alone. We know this to be the case, because in the brain slice preparation, STAT3 activation during neuroinflammatory responses is enhanced by the addition of proinflammatory cytokines and is blocked by neutralizing antibodies to proinflammatory cytokines (Damiani and O'Callaghan 2007). While activated STAT3 is required for the induction of astrogliosis (Herrmann *et al*. 2008; O'Callaghan *et al*. 2014), activation of the STAT3 pathway also occurs in response to exposure to the known inflammagen, lipopolysaccharide, in the absence of astrogliosis, likely through activation of microglia (O'Callaghan *et al*. 2014). These observations suggest that the neuroinflammation‐related activation of the STAT3 pathway after DFP and CPO results from inflammatory signaling through microglia.

Taken together, our findings show that exposure to CORT at levels associated with high physiological stress prior to acute administration of the GW‐relevant chemicals, DFP and CPO, results in a robust neuroinflammatory response that can serve as the basis of sickness behavior‐like symptoms associated with GWI. These effects are not related to the AChE inhibition induced by these agents. Both DFP and CPO, unlike PB and PHY, are OP compounds. It is possible that DFP and CPO exert their effects on the brain through the ‘organophosphorylation’ of certain neuroimmune targets, such as the JAK/STAT3 pathway or the phosphorylation and dysregulation of yet to be identified signaling pathways/substrates. While further research is needed to evaluate the role of phosphorylation in the neuroimmune system relevant to GWI, several studies have documented the role of protein phosphorylation in OP‐induced neuropathy (Abou‐Donia *et al*. 1988; Choudhary *et al*. 2001; Flaskos 2014). Thus, our findings may open a new avenue for discovery of therapeutic targets and treatments for GWI based on phosphoprotein profiling of signaling pathways (Zhu *et al*. 2010) involved in neuroimmune responses. Our data also demonstrate the potential for physiological stress to not only serve as a factor contributing to GWI, but also to play a role in exacerbation of chronic inflammatory disorders.
